# The harmonization of global professional certifications in emergency management: a narrative review

**DOI:** 10.3389/fpubh.2026.1894387

**Published:** 2026-09-17

**Authors:** Kyoo-Man Ha

**Affiliations:** Faculty of Resilience, Rabdan Academy, Abu Dhabi, United Arab Emirates

**Keywords:** global harmonization, global professional certification, host nation, multiple nations, stakeholders

## Abstract

**Introduction:**

This study aimed to examine methods for improving global professional certifications to achieve the ultimate goals of emergency management, such as reducing human casualties, economic damages, and psychological impacts.

**Methods:**

The research question, “How have global professional certifications in emergency management been harmonized?” was addressed through a narrative review as the primary methodology.

**Results:**

The study examined host nation-oriented global professional certifications and advocated for a shift toward multi-nation-oriented certifications, considering four key stakeholders: the United Nations Office for Disaster Risk Reduction (UNDRR), international non-governmental organizations (INGOs), emergency management agencies, and certification applicants.

**Discussion:**

Four stakeholders should work to transform host-nation-oriented certifications into multi-nation-oriented global professional certifications.

## 1 Introduction

International emergency management has expanded across multiple regions over the past three to four decades, leading to a growing demand for global professional certifications (1, 2). Initially, emergency managers were primarily first responders, such as firefighters, police officers, emergency medical personnel, and civil engineers. However, different nations have developed their own approaches to emergency coordination. In this context, the appropriate content for global professional certifications has been a topic of ongoing discussion, with a strong emphasis on professionalism.

Major information on global professional certifications is listed in Table 1; however, precise data on emergency management certifications are not yet available. By 2029, the professional certification market is expected to generate approximately USD 8.92 billion in revenue, up from USD 6.04 billion in 2024. In 2024, each certification candidate earned an average of USD 115. Among countries, the United States (US) generated the highest revenue that year, estimated at USD 1.956 billion, followed by China, Japan, India, the United Kingdom (UK), and other markets (3).

**Table 1:** List of major global professional certificates for emergency management.

Units		Requirements	Hosting organizations	Host nations
Certified emergency manager (CEM)		• Undergraduate diploma • 120 multiple-choice questions (minimum 75% answers) • Training • Work experience (three years) • Professional contributions (63). ^*^Canada, Oceania, and other regions have attempted to incorporate regional needs into these requirements.	International association of emergency managers (IAEM)	US
Certificate of business continuity institute (CBCI)		• 24-h (6-week) online course • 90 multiple-choice questions (minimum 70% correct) (64).	Business Continuity Institute (BCI)	UK
Certified emergency services manager (CESM)		• Written evidence of the candidate's work (3 years) in emergency services (65).	Australasian Institute of Emergency Services (AIES)	Australia
Etc.	Certified Business Continuity Professional (CBCP)	• Experience, multiple-choice exams, and online applications, among other requirements (66).	• Disaster Recovery Institute (DRI)International	US
	TIEMS Qualification Certification (TQC) and TIEMS Qualification Assessment Certification (TQAC)	• Experience, knowledge, and competence, among other criteria (67).	• The International Emergency Management Society (TIEMS)	Europe

An increasing number of researchers are addressing urgent topics in emergency management; however, few comprehensive studies have examined global professional certifications (4). Additionally, the effective adoption of global professional certifications has yet to be achieved (see Table 1). This is partly due to insufficient standardization, conformance, and interoperability among countries (5). A global emergency management certification ensures consistency in competencies, enabling professionals to respond efficiently to cross-border disasters. Such certification enhances emergency managers' mobility by supplementing local qualifications with internationally recognized skills. The research question is: “How have global professional certifications in emergency management been harmonized?”

The aforementioned professional certifications were chosen primarily because they are among the most well-established and globally recognized certification systems, as identified through online and documentary research. Specifically, three certifications—CEM, CBCI, and CESM—were chosen prior to the review process and functioned as the predetermined units throughout the investigation. Since the number of global professional credentials continues to grow and including all was beyond the scope of this review, additional certifications were excluded (6, 7). Given that the chosen certifications are extensively used in professional practice and reflect significant emergency management traditions across many areas, these options are unlikely to compromise the study's representativeness.

This study investigated methods to improve the issuance of global professional certificates in the field of emergency management to achieve global harmonization. Four analytical units—the UNDRR, INGOs, emergency management agencies, and certificate applicants—analyzed host-nation-oriented global professional certification to develop a multi-nation-oriented global professional certification framework. The impact of this multi-nation-oriented certification is supported throughout the manuscript.

## 2 Literature review

### 2.1 Basic concepts

Emergency management involves addressing natural hazards and man-made emergencies to ensure public safety through collaboration with private companies, voluntary organizations, and other local stakeholders (8). Within the emergency management cycle, preparedness includes emergency training, which may involve internationally recognized professional certification. Unlike education, emergency training typically consists of a brief sequence of related courses and a high degree of specialization and does not contribute credit toward a degree or diploma.

Professionals engage in related activities as paid occupations that require formal qualifications and prolonged training (9). They typically hold a college degree, possess extensive knowledge, demonstrate strong performance abilities, receive professional recognition, make ethical decisions, and show a deep commitment to their field. Similarly, in emergency management, professionalism denotes the level of competence and practice expected from professionals.

Professional certifications are credentials that individuals can earn to validate their skills in emergency management. Global professional certifications are not only applicable in a specific region but anywhere in the world (10). These certifications establish an internationally recognized standard of knowledge and skills, promoting cross-border coordination and interoperability during disasters, particularly for international emergency managers. However, they should also allow for local adaptations to address region-specific challenges while adhering to key emergency management principles. This study included certifications written and administered in English due to the scope of the research. While supplementing emergency management education and professional experience, global professional certifications play a crucial role in preparing professionals for critical emergencies.

Accreditation refers to the official recognition that global professional certifications meet established global standards across various aspects of emergency management. It plays a crucial role in enhancing the quality of these certifications (11). In the US, the Emergency Management Accreditation Program (EMAP) recognizes programs conducted by service delivery organizations, such as state and municipal governments and hospitals. Accreditation institutions in various regions strive to achieve their objectives by establishing emergency management standards, conducting rigorous assessments, and offering other quality assurance programs. Consequently, accreditation is widely advocated for ensuring quality assurance, certification reliability, regulatory compliance, and public trust.

### 2.2 Previous studies

Although professional certifications have been studied across various fields—including education, vocational training, health sciences, and tourism—research on global professional certifications in emergency management remains limited (12, 13). Some researchers and practitioners in emergency management have expressed interest in investigating the formation and subsequent issuance of professional certificates within their field (14). However, this study warrants significant attention, as comprehensive studies in this area are scarce.

Galbraith and Gilley (15) argued that a profession should evaluate practitioners' proficiency through relevant certification. They maintained that professional certification was essentially based on voluntary service. In summary, the certification process was primarily regulated by the profession itself rather than by external organizations, as it contributed to human resource development and education. Although the process had drawbacks, such as financial costs, fragmentation, and legal challenges, it also offered benefits, including standardization, professional contributions, and enhanced recognition.

Somers and Svara (16) warned that issuing excessive certificates in emergency management could lead to the emergence of certificate mills. While investigating the close connection between traditional local government administration and the relatively new field of emergency management, the authors partially explored certification in the industry. When local governments grant emergency training certificates to trainees without adequate evaluation, the result is substandard emergency management practices. Therefore, researchers emphasize the necessity of rigorous certification standards.

Waugh and Sadiq (17) discussed methods for training American emergency managers by referring to the CEM program. They noted that the certificate's content was not fully balanced. Similarly, the CEM aims to integrate emergency management theories and practices, fundamental professional values, and organizational relationships. However, it does not sufficiently emphasize the four phases of the emergency management cycle, emergency support functions, or the characteristics of specific hazards.

While acknowledging that education and training play a key role in managing local emergencies, Ingrassia et al. (18) noted that the European Union lacked standardized training programs across its member states. Among the many self-funded training initiatives, most provide multidisciplinary content to emergency trainees. However, competency-based training frameworks often fail to incorporate essential components of emergency management into their curricula. Studies strongly indicate the need to develop standardized training programs.

Ruiz-Molina et al. (19) studied the value of associated certifications by surveying respondents about European certification programs. They found that the primary value of a certification depends on both the applicants' characteristics and the certification schemes. These schemes include future applicability (the most important attribute), fairness and difficulty, affordability, innovative aspects, credibility, international acceptance, and more. When a certification is perceived as highly useful in the near future, many candidates are eager to obtain it.

Dippy (20) argued in favor of certifying emergency management workers in Australia. The impacts of climate change—including bushfires, droughts, coastal erosion, and floods—and the Coronavirus Disease of 2019 (COVID-19) pandemic have severely affected the country. It was believed that Australia could not effectively manage these disasters without strengthening human capabilities, including those of police, emergency responders, multi-agency teams, and foreign colleagues. Dippy (20) concluded that developing human capabilities should be grounded in an adequate certification process.

### 2.3 Premise of this study

This study largely upholds the rationale for global harmonization, considering various associated issues such as global professional certifications, international emergency management, diverse nations, and multiple interests (21). The goal is to enable comparisons across different certification systems while highlighting the importance of global standards. Additionally, the discussion differentiates among global, national, and local certifications. Global harmonization involves aligning individual professional certifications worldwide through standardized principles, universal guidelines, global communication, and shared interests to support effective international emergency management. Without global harmonization, many global professional certifications may not be applicable to candidates in different regions.

This study identified two distinct approaches to global professional certification: host-nation-oriented and multi-nation-oriented. The former refers to existing international professional certificates grounded in the knowledge and practices of the host nation, including emergency terminology, emergency management principles, organizational mechanisms, and emergency culture (22). The latter envisions future global professional certificates that incorporate knowledge and practices from multiple countries (23). Regarding global harmonization, these two approaches are opposed, representing negative and positive perspectives, respectively.

## 3 Methods

### 3.1 Analysis design

Figure 1 presents the analytical framework used in this study. While the study acknowledges its efforts to collect, analyze, and interpret both manifest and latent qualitative content related to global professional certificates, its primary methodology is classified as a narrative review (24). Instead of statistically aggregating empirical findings, the study aims to analyze, synthesize, and compare various sources of data on international professional certifications in emergency management. Therefore, a narrative review was deemed appropriate (25).

**Figure 1:**
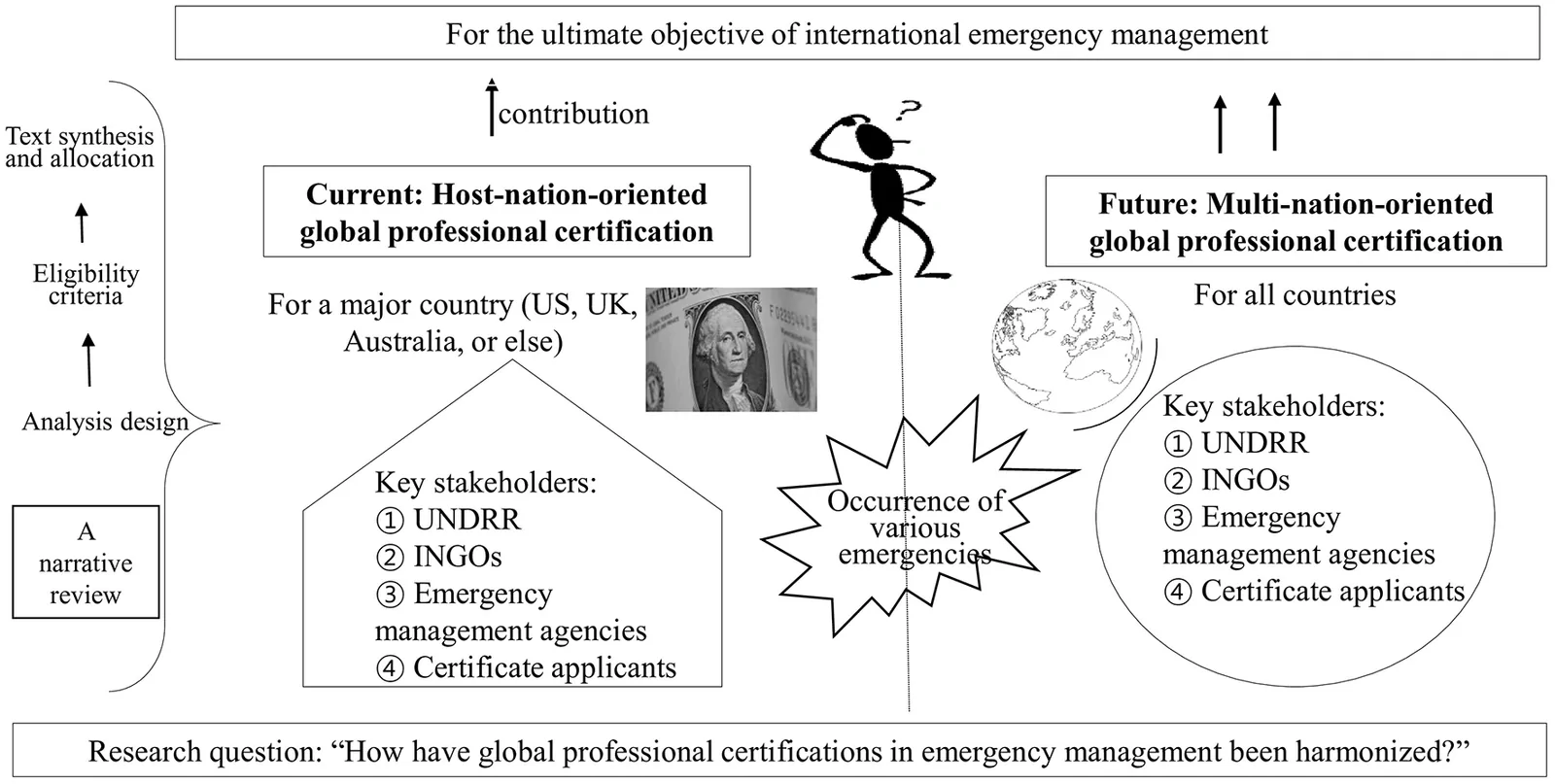
Analytical framework. *Source*: Created by the author.

Although this study was not designed as a systematic review, the Preferred Reporting Items for Systematic Reviews and Meta-Analyses (PRISMA) 2020 flow diagram was used to document the processes of literature identification, screening, eligibility assessment, and inclusion (see Figure 2). Duplicate records were eliminated where appropriate. After screening titles and abstracts, possibly eligible publications were reviewed in full, thus enhancing the transparency of the narrative review. To determine the key traits, parallels, discrepancies, and potential future paths of international professional certification systems, the collected literature was then synthesized through narrative interpretation (26).

**Figure 2:**
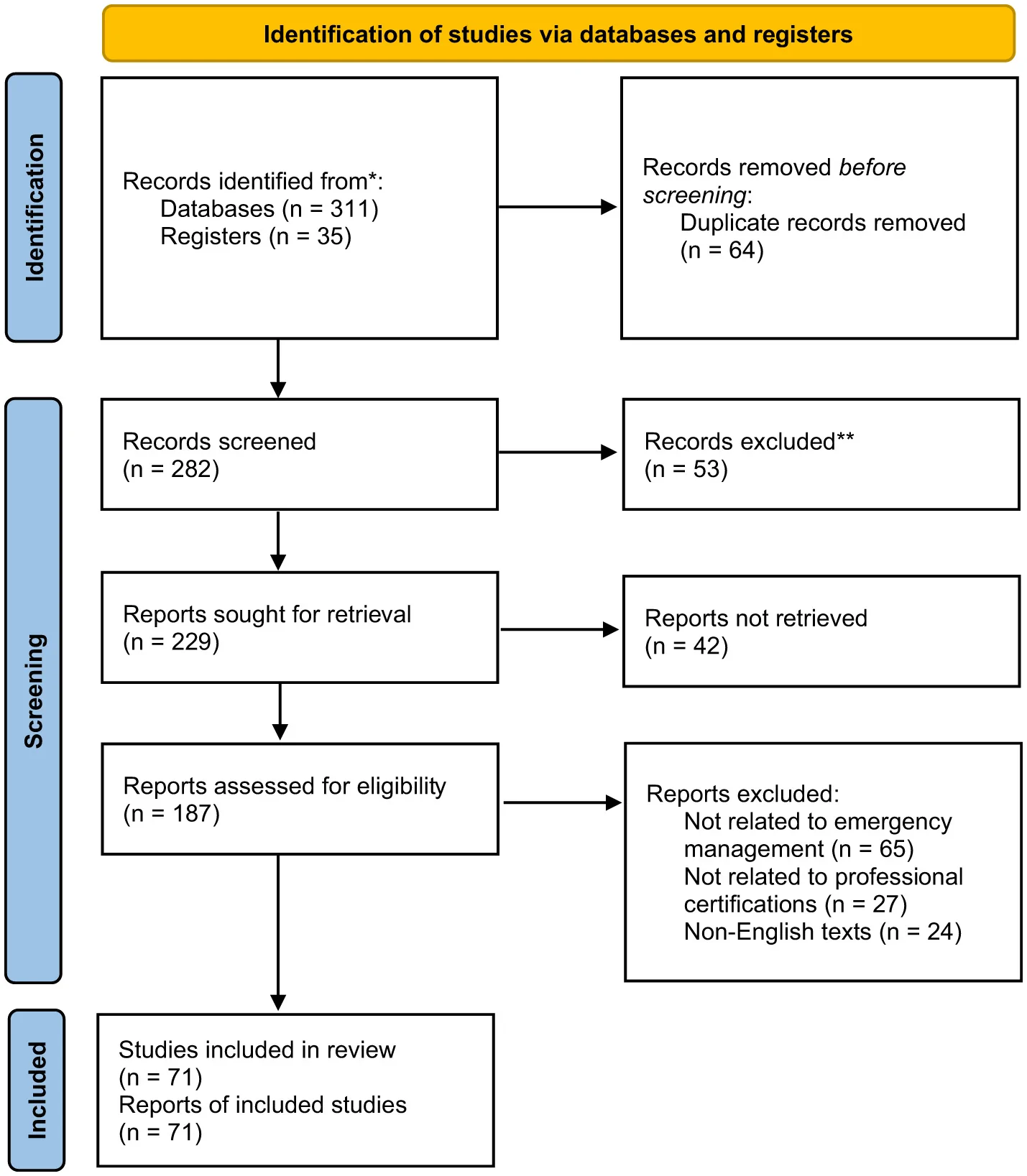
PRISMA 2020 flow diagram. *Source*: Page et al. (62). *Consider, if feasible to do so, reporting the number of records identified from each database or register searched (rather than the total number across all databases/registers). **If automation tools were used, indicate how many records were excluded by a human and how many were excluded by automation tools. *Source*: Page et al. (62). This work is licensed under CC BY 4.0. To view a copy of this license, visit https://creativecommons.org/licenses/by/4.0/.

### 3.2 Eligibility criteria

Assuming that many countries have attempted to provide professional certificates in emergency management (27), a major criterion for text inclusion or exclusion was whether the materials, including the content of professional certificates, were written in English. French-speaking regions such as France, Monaco, and Quebec in Canada offer professional certificates in French, while the Arab Institute for Continuing Professional Development in Cairo, Egypt, issues certificates in Arabic. Only texts published in English were included in this analysis. Studies or official documents pertaining to professional certifications in emergency or disaster management constituted the second inclusion criterion. Due to the limited number of rigorous academic studies on this topic, the third inclusion criterion permitted the use of a small number of gray literature sources, such as organizational websites and other non-traditional publications. Most of these gray literature sources were legitimate websites created by organizations responsible for creating, managing, or upholding professional certification programs. In contrast, exclusion criteria encompassed non-English publications, duplicate records, editorials lacking substantive discussion, unrelated publications, and papers with insufficient information for narrative synthesis.

The search terms used included “emergency management certification,” “professional certification,” “disaster management certification,” “certificates and certification,” “professional certificates and disaster management,” and “global professional certification.” Boolean operators (AND, OR) were applied to combine keywords, for example: (“emergency management” OR “disaster management”) AND (“professional certification” OR “certification”). To increase the scope of the literature search while maintaining relevance to the research topic, synonyms and variations were also considered.

Following the identification of appropriate English texts, this study applied several eligibility criteria. Whenever possible, the most recent texts were prioritized as a search filter. However, older materials were also included when discussing the history of professional certificates. The primary focus was on research articles, although books and government documents were also considered (28). The study initially began with Google Scholar but was significantly expanded to include Scopus, ScienceDirect, EBSCOhost, IEEE Xplore, Wiley Online Library, Web of Science Core Collection, and other relevant databases to capture a broad range of academic and professional sources. Between January 2025 and August 2026, the search was carried out by utilizing the above databases.

The search procedure was designed to achieve extensive and balanced coverage of the issue rather than exhaustive retrieval of every possibly eligible publication, as this study used a narrative review approach (29). Consequently, the eligibility requirements remained sufficiently flexible to accommodate various publication formats and new topics pertinent to international professional certification in emergency management.

### 3.3 Text synthesis and allocation

Since text synthesis involves combining and evaluating extracted texts (30), this study divides the analysis into two categories: host-nation-oriented global professional certification and multi-nation-oriented global professional certification. The former represents the current reality of worldwide professional certification, while the latter is presented as a potential future option. For text allocation, this study maintains the same four analytical units—UNDRR, INGOs, emergency management agencies, and certificate applicants—across both themes. Using these consistent units facilitates a systematic comparison between the two analytical themes. By analyzing the viewpoints and duties of important stakeholders in both certification processes, this thematic structure enables a more organized and coherent comparison of the literature (31).

The four selected units aim to incorporate various significant stakeholders into the discussion of global professional certifications. These stakeholder groups were chosen because they offer a comprehensive viewpoint at both national and international levels and represent the key players in the creation, application, governance, and use of global professional certifications in emergency management. While the UNDRR is a prominent global agency for international emergency management, INGOs are responsible for providing and maintaining many global professional certifications. In response to demands from certificate applicants, national emergency management agencies have established requirements for worldwide certification (32, 33). The first two units represent international stakeholders, while the remaining two represent national stakeholders.

Employing the same analytical approach, the narrative analysis facilitates the comparison of each stakeholder group's roles, responsibilities, contributions, opportunities, and problems in advancing internationally recognized professional certification. Similarly, to identify common patterns, differences, obstacles, and opportunities for developing globally accepted professional certification systems, the narrative synthesis integrates information from these stakeholders.

The narrative review used a structured qualitative coding procedure to increase methodological transparency (34). Significant sentences pertaining to international professional certifications in emergency management were extracted and given initial descriptive codes after pertinent texts found using the eligibility criteria were read several times. Rather than applying a predefined coding framework, the coding method was mostly inductive, allowing patterns to arise naturally from the examined texts. To ensure analytical consistency throughout the review process, a single reviewer completed all coding. Two coworkers independently examined the coding system, offering peer review and verifying that the interpretations accurately matched the retrieved literature, thereby increasing the review's trustworthiness. The two themes (or categories) were considered final when they offered a coherent analytical framework for comparing the four stakeholders and fully reflected the key patterns seen across the examined texts.

## 4 Results: host-nation-oriented global professional certification

### 4.1 UNDRR

Since risk management is everyone's responsibility, the UNDRR has conducted workshops (in-person training) and webinars (online training) worldwide, leading to global professional certifications (35). For example, the Global Education and Training Institute (GETI) in South Korea, under the UNDRR, has collaborated with governments, businesses, and voluntary organizations globally. Additionally, the UNDRR's Making Cities Resilient campaign has facilitated the exchange of information on resilience capacities and best practices with national training organizations through professional certifications. Although the implementation of these programs has mostly mirrored host nation settings rather than globally unified certification standards, the analyzed documents indicate that they have contributed to professional capacity development across several areas.

Despite acknowledging the importance of various geographical settings and local cultures, the UNDRR's global professional certifications have frequently focused on nation-specific examples and related experiences (36). Since it highlights excellence in international emergency management, GETI has primarily adopted a nation-specific strategy. Specific examples include capacity-building training in Italy (2015), urban resilience initiatives in the Philippines (2017), disaster risk management efforts in Nepal (2019), and interregional discourse in the Asia-Pacific region (2024).

### 4.2 INGOs

The IAEM, headquartered in Falls Church, Virginia, recently introduced two global professional certifications: Associate Emergency Manager (AEM) and CEM. However, its primary focus has remained on the US emergency management system (37). As an INGO, the IAEM has developed a mutually supportive relationship with the Federal Emergency Management Agency (FEMA). Consequently, it has integrated various government courses into its global professional certification programs. The IAEM shares the same emergency management goals as the US government. Rather than demonstrating that one method is superior to another, the literature consistently characterizes this relationship as a distinguishing feature of the current certification scheme.

Other INGOs have issued professional certifications worldwide; however, each certification is heavily influenced by the host country's emergency management policies and strategies (38). Similarly, BCI (UK) classifies business continuity into strategic, tactical, and operational levels, whereas IAEM (US) classifies it sequentially as strategic, operational, and tactical. AIES emphasizes that the most important aspect of its CESM requirements is individuals' ongoing professional growth—including skills and knowledge—while adhering to Australian national values.

### 4.3 Emergency management agencies

FEMA has recently updated its strategies for global professional certification; however, its focus remains rooted in conventional American values. Consequently, FEMA continues to provide financial support for the IAEM's emergency training initiatives. Additionally, FEMA has granted foreign nationals access to the Emergency Management Institute's (EMI) Independent Study program, which now offers nearly 130 courses (39). While fundamental components such as the Incident Command System (ICS) and local government autonomy have been successful within the US, they are not universally applicable to other countries.

Governments in other nations have undertaken initiatives related to global professional certifications while considering their national interests (40). The UK government has indirectly supported the BCI (as an NGO) and the CBCI. UK strategies include establishing regulatory standards, collaborating with the BCI, and recognizing the CBCI within the public sector. Similarly, some members of the Australian government have attempted to support initiatives related to the AIES and CESM. However, these independent efforts have not yet been fully realized in practice. With relatively little evidence of globally coordinated certification frameworks, the reviewed literature typically indicates that national governments have supported certification programs within their respective institutional and policy environments.

### 4.4 Certificate applicants

When admitting that many certificate applicants benefit from cultural familiarity, it is important to acknowledge that not everyone worldwide can navigate and complete a designated certification process (41). Applicants with an acceptable educational, social, or cultural background in the host nation of a specific certification often find the process easier. For example, American citizens or foreigners who have studied in the US have a higher likelihood of obtaining the CEM certification. In contrast, foreigners with no prior experience in the UK or Australia may face greater challenges in meeting the requirements for the CBCI or CESM certifications.

Similarly, the issue of English has posed difficulties for many non-native speakers, particularly due to its gatekeeping effect (42). While it is true that many applicants should earn English certificates as a mark of achievement, the requirement that all global professional certification processes be conducted solely in English may discourage non-English-speaking candidates from applying. Furthermore, candidates with limited English proficiency often struggle to pass the associated exams. Language barriers are a significant concern, as global professional certifications are intended to assess candidates' understanding of emergency management rather than their English proficiency. These results suggest that linguistic and cultural influences are recurrent themes found in the literature and should be interpreted as descriptive observations rather than normative judgments.

## 5 Discussion

### 5.1 Multi-nation-oriented global professional certification

The issue of global professional certification is considered a strong foundation in emergency management, as obtaining an internationally recognized certificate is one of the most important measures of an individual's competency in this field (43). By completing the certification process in complex emergency management, certificate holders can enhance their knowledge and practical skills. This, in turn, enables them to contribute effectively to the improvement of both their organizations and local communities (44).

Further promotion of global professional certifications may help reduce human casualties, economic losses, and psychological stress caused by multiple emergencies (45, 46). When stakeholders manage risks more systematically, the physical impacts of emergencies—such as human casualties and economic damage—may decrease. Social impacts, including inequality and psychological distress, are also likely to be reduced across regions. Strengthening the rationale for global professional certification may support international emergency management goals.

Since a significant portion of professionals in the field are classified as emergency managers, the certificate is issued to support their development across various locations (47, 48). Emergency managers strive to understand natural hazards and environmental changes, man-made emergencies and social shifts, technological hazards, and engineering challenges, among other areas, while coordinating complex issues. Certification procedures provide emergency managers valuable learning opportunities to enhance their job performance.

Many actors involved in global professional issues are connected to certified instructors and peers through networks (49). Since potential certificate holders join different networks after completing the certification process, this process may enhance professional networking. Even those who are not current certificate holders—such as incumbent certificate holders, government officials, international figures, and volunteers—may become part of new networks. The involvement of multiple actors may further accelerate professional development.

The principle of interoperability may be applied to global professional certifications, ensuring that individuals, institutions, systems, and even nations can collaborate effectively in emergency management (50). Key aspects include the standardization of emergency training, the use of technology for international credentialing, mutual recognition of certifications, and policy harmonization. An international framework for global certification (or interoperability) could be established across national borders.

Based on the research question, “How have global professional certifications in emergency management been harmonized?” the emergency management field is transitioning from host nation-oriented certification to multi-nation-oriented certification. Table 2 illustrates how key stakeholders can achieve the status of multi-nation-oriented global professional certification in response to the limitations of host nation-oriented certification. Those key strategies present the author's conceptual recommendations derived from the narrative synthesis. These recommendations are not direct findings from the reviewed literature but reflect an interpretive synthesis of the key patterns and gaps identified across the literature. In doing so, relying solely on host nation-oriented certification continues to disadvantage stakeholders by perpetuating inequities (51). These certifications lack fairness for multiple stakeholders, particularly individuals from diverse emergency environments, such as special-needs populations who may face additional barriers due to language differences. In response, host nations demonstrate their commitment to inclusivity by transitioning to multi-nation-oriented certification systems.

**Table 2:** Potential opportunities and alternatives for multi-nation-oriented global professional certification.

Analytical units	Key strategies
UNDRR	UNDRR could integrate GETI, the Making Cities Resilient campaign, and other initiatives to enhance the sharing of international emergency management information and best practices with member states and other stakeholders. This could be achieved through the creation of more regular and systematic platforms (68). GETI, in particular, may explore hybrid strategies beyond a nation-specific approach by incorporating diverse national examples and representations simultaneously.
INGOs	Despite ongoing efforts, IAEM could address various activities related to multilingual accessibility, including the localization of certification materials, the establishment of local language support teams, and the provision of multilingual exam options in addition to English-language exams. INGOs, such as BCI, IAEM, and AIES, must consider International Organization for Standardization (ISO) norms when addressing differences between themselves and other stakeholders (69).
Emergency management agencies	FEMA could further integrate non-American perspectives into its emergency management processes, including global professional certifications, by engaging international specialists and reviewing training materials in collaboration with indigenous communities worldwide (70). The UK and Australian governments could collaborate with—or lead—INGOs such as BCI and AIES, encouraging them to rigorously evaluate and revise CBCI exam questions, CESM requirements, and training materials from an international perspective.
Certificate applicants	Due to cultural unfamiliarity, eligible certification applicants could be encouraged to communicate their concerns to INGOs or other certification bodies and explore alternative options, such as customized evaluation methods, tailored exam materials, pilot programs, and mentorship opportunities. To overcome language barriers, certification applicants may seek language support from exam administration organizations, utilize translation tools, practice with mock or past exams, participate in joint study sessions, and explore other available resources (71).

Global harmonization of professional certifications presents several drawbacks despite its benefits (52). First, because emergency management systems vary greatly among nations in terms of institutional capacity, governance structures, legal authorities, and risk profiles, total harmonization is neither practical nor necessarily desirable. Second, practical obstacles to implementation exist, including financial expenses, organizational capacity limitations, and challenges in obtaining mutual acceptance among certification bodies. Third, the direct adoption of globally harmonized certification standards may be hampered by variations in professional culture, educational customs, administrative procedures, and country laws controlling professional qualifications. Therefore, stakeholders should adopt a flexible harmonization strategy rather than pursuing total standardization.

One of the most important managerial implications of the transitioning trajectory is cooperation (collaboration or coordination) among all stakeholders (53). Global professional certification involves complex processes spanning multiple countries. Therefore, all stakeholders must work together to integrate key components, minimize discrepancies, and advance the shared goal of emergency preparedness. Without a willingness to collaborate on long-term revisions, the initiative for multinational certification cannot be completed in a timely manner.

The transition to multi-nation-oriented certification may progress alongside the improved integration of theory into practice, as evidenced by the recognition that such integration aligns with the broader scope of global professional certifications (54). These two topics complement each other due to their interdependence. Specifically, both subjects fall within the context of emergency management and aim to achieve global-level theories, cross-border practices, unified frameworks, and related objectives through various approaches.

The issue of multiple certification providers must be considered when transitioning to internationally recognized certificates. These providers include various international organizations, national and municipal agencies, and other regional entities, all contributing to professional global certification (55). Multiple certification providers play a crucial role in facilitating this transition by ensuring diversity in certification standards, regional recognition, specialization, and overall accessibility. Therefore, mutual recognition and cooperation among certification providers may be essential for success.

Similarly, the transition could further enhance international standards by integrating common emergency management principles into global professional certifications (56). Universal standards—such as the disaster management cycle, risk management, business impact analysis, and lessons learned—may help ensure the quality of professional certifications and the comparability of emergency training programs. In the absence of international standards, stakeholders often engage in repeated consultations and support activities before developing new standards.

During this transition, the popularity of global professional certifications is expected to increase (57). Similarly, the US tourism sector has recently introduced crisis preparedness certificates, whose popularity has risen due to the immediate impact of COVID-19. In particular, the demand for multinational certification may grow rapidly in countries with shared emergency management interests, highlighting that cascading emergencies or complex disasters cannot be effectively managed by any single nation alone.

The current results (or transition) confirm previous findings, such as those by Larson and O'Toole (14) and Galbraith and Gilley (15), which identified professional certification as a crucial tool for enhancing emergency management competency. However, this study advances earlier research by demonstrating that the value of certification should now be viewed primarily from a multinational perspective that prioritizes interoperability, mutual recognition, and international inclusivity rather than from a single national viewpoint. The latest findings expand the conversation by offering a framework for standardizing certification across nations, whereas earlier research focused solely on the importance and quality of certification systems.

Additionally, the results build upon the research of Waugh and Sadiq (17), Ingrassia et al. (18), and Dippy (20). While their main focus was on enhancing national workforce capability, competency-based training, and certification materials, the current study argues that achieving internationally recognized certification requires subsequent cross-border collaboration among four key stakeholders. This broader governance perspective emphasizes international collaboration as essential for global professional certification, extending beyond curriculum development.

The transition partially supports the findings of Ruiz-Molina et al. (19), who noted that future applicability and global acceptance are crucial characteristics of certification programs. This study further expands on their findings by asserting that worldwide acceptance depends not only on applicants' perceptions but also on institutional cooperation, multilingual accessibility, policy harmonization, and interoperability among certification providers. Unlike Somers and Svara's (16) concerns about certificate proliferation and certificate mills, this study does not advocate issuing more certificates. Instead, it suggests enhancing the quality, mutual acceptance, and global uniformity of existing certification systems.

### 5.2 Strengths and Limitations

By recognizing the evident gap between research on emergency management and research on global professional certifications (58), this study aims to bridge that divide. Given the increasing demand for global professional certifications in this field, it serves as a theoretical reference for future research. Specifically, it provides a foundational understanding of the theoretical framework surrounding professional credentials worldwide and offers a comprehensive perspective on the purpose of emergency management.

This study's focus on international harmonization in emergency management is distinctive. By considering the diverse needs, priorities, and professional contexts of various nations in connection to internationally recognized emergency management certifications, the study adopts a global approach rather than advancing the interests of any single country (59). It aims to identify universal concepts and shared expectations that can enhance the professional competence of emergency managers worldwide, rather than endorsing any specific national certification system or institutional approach.

However, due to space constraints, this study primarily examines the features of three global professional certificates—CEM, CBCI, and CESM—within the theoretical framework. It does not address global certifications offered in other languages, such as French, Spanish, Arabic, or Chinese. One limitation is the potential lack of representative samples, as selection may have been influenced by individual criteria (60). Consequently, other global or regional professional certificates relevant to emergency management may have been omitted because they did not meet the study's predefined selection criteria.

Despite employing predetermined search strategies and selection criteria, personal judgment may have influenced the identification, classification, and interpretation of the included literature (61). Document selection, eligibility assessment, data extraction, and thematic analysis were primarily conducted by a single investigator. Although efforts were made to ensure consistency and transparency throughout the review process, some pertinent publications or alternative theme interpretations may have been overlooked due to researcher subjectivity.

## 6 Conclusion

This study aimed to explore ways to enhance the category of global professional certifications in emergency management by addressing the research question: “How have global professional certifications in emergency management been harmonized?” We examined fundamental concepts, previous studies, four analytical units (the UNDRR, INGOs, emergency management agencies, and certificate applicants), major consequences, and other relevant factors. Notably, the findings suggest that this study was achieved by comparing host nation-oriented global professional certifications with multi-nation-oriented global professional certifications.

The main theme of this study is that all four stakeholders must collaborate to transform host-nation-oriented certification into a multi-nation-oriented certification system aimed at achieving equity. Each stakeholder should sequentially implement the proposed actions, which include establishing a systematic platform, expanding multilingual support, incorporating non-American perspectives, addressing cultural barriers, and more. They should also continue to enhance individual competencies, foster professional networking, improve interoperability, promote international cooperation and integration, engage multiple certification providers, support standardization, and increase certificate recognition.

Using the findings of this study as a guide, scholars can further explore the subject of global professional certifications in emergency management. Numerous themes are available for exploration, including regional case studies, cost-benefit analyses of professional credentials worldwide, and technological advancements in multilingual examinations. To address the limitations of this study, researchers should consider various professional certifications and incorporate multiple languages in their analyses. Global professional certifications are expected to become increasingly important in the field due to their associated activities.

## Data Availability

The original contributions presented in the study are included in the article/supplementary material, further inquiries can be directed to the corresponding author.
